# Possibility of AnteOwl IVUS–based antegrade dissection and reentry using the tip detection method for CTO-PCI

**DOI:** 10.1007/s12928-022-00846-2

**Published:** 2022-02-15

**Authors:** Atsunori Okamura, Hiroyuki Nagai, Kota Tanaka, Satoshi Suzuki, Heitaro Watanabe, Katsuomi Iwakura

**Affiliations:** Cardiovascular Center, Division of Cardiology, Sakurabashi Watanabe Hospital, 2-4-32 Umeda, Kita-ku, Osaka, 530-0001 Japan

**Keywords:** Coronary intervention, Chronic total occlusion, IVUS-based ADR, Tip detection method

In chronic total occlusion (CTO) intervention, it has been determined that it is impossible to create a reentry from a subintima to a true lumen with regular guidewire manipulation. Therefore, antegrade dissection and reentry (ADR) using Stingray system (Boston Scientific, Natick, MA, USA) was developed. In the present case, ADR using a Stingray balloon failed to create a reentry. Intravascular ultrasound (IVUS) observation revealed the damaged part of the wall by ADR. We could create a reentry at the damaged part by an exact vertical directional puncture using the tip detection method with an AnteOwl WR-IVUS (AO-IVUS; Terumo Corp., Tokyo, Japan) [1].

A 74-year-old man suffered from effort angina pectoris due to a CTO lesion in the left anterior descending coronary artery because of occlusion of bypass grafting to it. Computed tomography and angiography showed severe calcification in the CTO (Fig. 1A, B). An 8 Fr guide catheter was inserted from the femoral artery. Any guidewires including a Confianza-20 g (Asahi Intecc Co., Ltd., Aichi, Japan) could not enter the severe calcified lesion, but a XT-A wire (Asahi Intecc) supported by over-the-wire balloon anchoring could be advanced through the subintimal space using the knuckle wiring. We moved on to angiography-based ADR using a Stingray balloon. Using Confianza-20 g and XT-R wires, five attempts of the stick-and-swap technique were performed, but the guidewire could not be led into the true lumen which was not visible by angiography. AO-IVUS observation revealed the somewhat damaged part of the true lumen wall which might have been caused by the punctures during ADR 6 mm beyond the CTO exit (Fig. 1E). A vertical puncture might not be possible by angiography-based ADR because it depends on fluoroscopy. We moved onto AO-IVUS-based ADR using the tip detection method to perform an exact vertical directional puncture at the damaged part (Fig. 1C). A GAIA Next 3 supported by a Corsair (Asahi Intecc) was used because penetration force would be required for reentry. The tip detection method allowed the tip of the wire to puncture the wall of the true lumen in an exactly vertical direction, resulting in the successful reentry (Fig. 1F–H, ESM Video 1). Normal antegrade blood flow was achieved after stenting (Fig. 1D). Going forward, if guidewires with more penetrating force are used, they may allow for intentional reentry even for the undamaged walls using the AO-IVUS-based tip detection method.

**Fig. 1:**
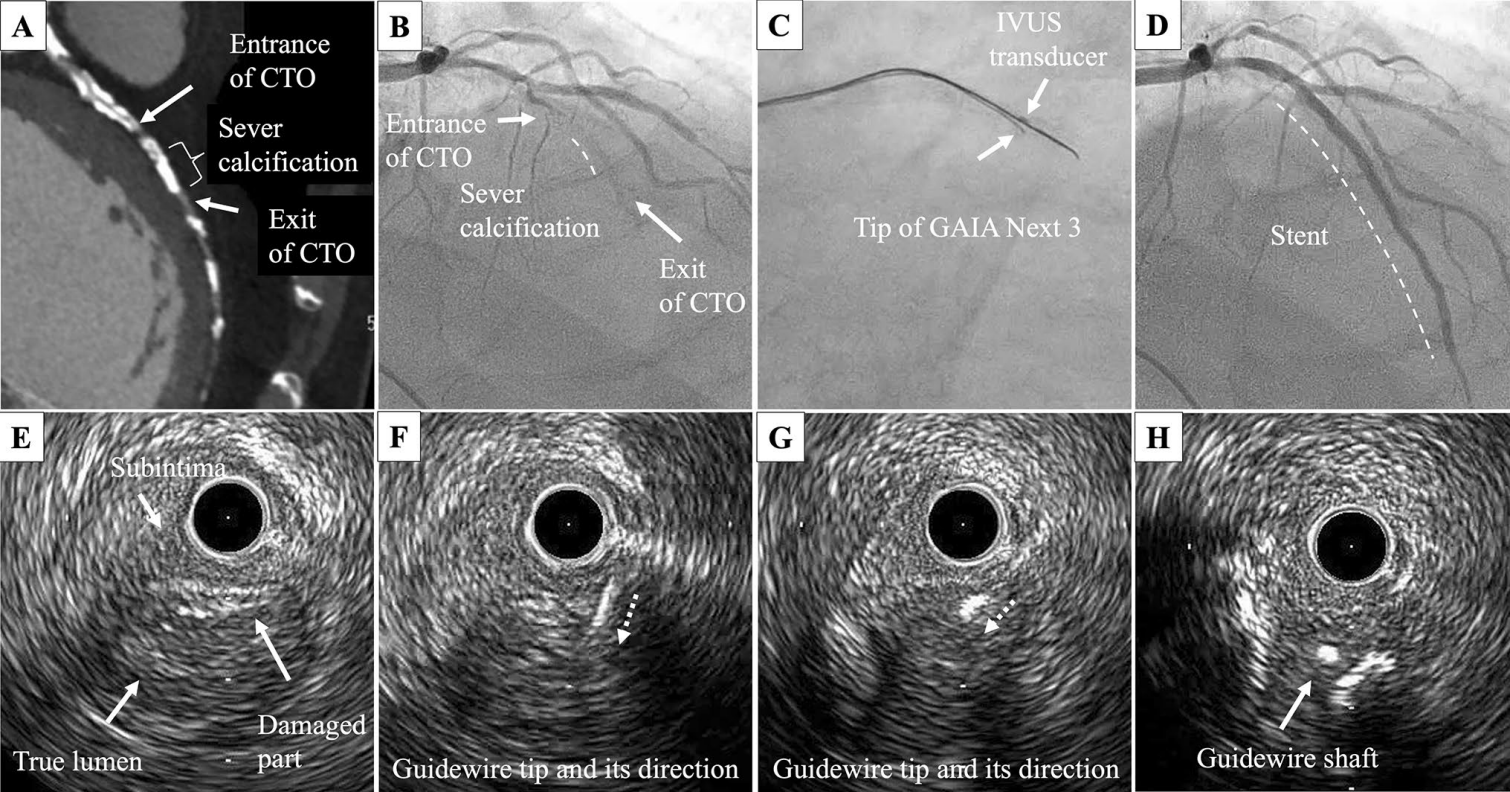
Computed tomographic image (**A**), angiographic images (**B**) prior to, **C** during, and **D** after the procedure, and intravascular ultrasound images **E** prior to, **F**, **G** during the puncture, and **H** after successful puncture. *CTO* chronic total occlusion

## Supplementary Information

Below is the link to the electronic supplementary material.

Supplementary file1 Intravascular ultrasound images during the puncture of the true lumen using the tip detection method. (MP4 22361 KB)

## Abbreviations

ADR: Antegrade dissection and reentry
AO-IVUS: AnteOwl WR-intravascular ultrasound
CTO: Chronic total occlusion
IVUS: Intravascular ultrasound
